# Supplementary material for: Levels and predictors of anxiety, depression, and stress during COVID-19 pandemic among frontline healthcare providers in Gurage zonal public hospitals, Southwest Ethiopia, 2020: A multicenter cross-sectional study
Source: PLoS One. 2021 Nov 29;16(11):e0259906. doi: 10.1371/journal.pone.0259906 (PMC8629282; doi:10.1371/journal.pone.0259906)
Supplement: Attachment — Submitted filename: Response to Reviewers.docx [file pone.0259906.s003.docx]

**To:** PLOS ONE

**Subject**: *Replay to Review Report* #2

**Manuscript Ref. No**: [PONE-D-21-20584] - [EMID:528479536e1a8b26]

**Manuscript title**: *“****Levels and predictors of anxiety, depression, and stress during COVID-19 pandemic among frontline healthcare providers in Gurage zonal public hospitals, Southwest Ethiopia, 2020: A Multicenter Cross-Sectional Study:****”*

**Date:** *October 26/ 2021*

**Authors:**

*Fisha Alebel GebreEyesus^*^ ^1^, Tadesse Tsehay Tarekegn^1^ , Baye Tsegaye Amlak ^1^, Bisrat Zeleke Shiferaw^1^ , Mamo Solomon Emeria ^1^, Omega Tolessa Geleta ^1^ Tamene Fetene Terefe ^1^, Mtiku Mamo^2^, Melkamu Senbeta Jimma ^3^, Fatuma Seid Degu^14^, Elias Nigusu Abdisa^5^ , Menen Amare Eshetu^6^, Natnael Moges Misganaw^7^, Ermias Sisay Chanie ^7^*

**Dear Reviewers and editors:**

**Greetings of the day!**

First of all, we would like to present our gratitude acknowledgment, and appreciation for the effort you made to improve our manuscript throughout the review process during this difficult time of the COVID-19 pandemic. Also, we wish to thank you for considering this manuscript for publication in your journal.

Dear Reviewers and editors, on behalf of the authors I am submitting the revised version of the manuscript. We have gone through your constructive comments and question and devote all our effort to incorporate the feedback. All the authors are grateful to the reviewers and editors for their candid comments and timely communication.

All the essential revisions are incorporated in the main manuscript and next to this cover letter please, kindly have a point-by-point guide on the response given to the comments/concerns and questions raised by reviewer # 1.

***Sincerely,***

***Fisha Alebel GebreEyesus (MSc)*** *- (corresponding author) on behalf of all authors)*

**Response to Reviewer#1**

***Dear reviewer,***

First of all, we would like to present our gratitude acknowledgment, and appreciation for the effort you made to improve our manuscript throughout the review process during this difficult time of the COVID-19 pandemic. We also would like to express our heartfelt gratitude for your candid comments which we have addressed to the best of our abilities to improve the quality of our manuscript.

***Review Comments to the Author***

Please use the space provided to explain your answers to the questions above. You may also include additional comments for the author, including concerns about dual publication, research ethics, or publication ethics. (Please upload your review as an attachment if it exceeds 20,000 characters)

Reviewer #1: The authors have addressed the most of the issues except for the following.

They still mistakes “odds ratio” for “relative risk”. “Odds ratio = 2.0” dose not mean “two times more likely”. We can say “two times more likely” only when the relative risk is 2.0 in a prospective cohort study. As this study is a cross-sectional case-control studies, they cannot mention the relative risks. The odds ratio can be occasionally used as an approximation of the relative risk when patients with diseases are less than 1% in general. However, the odds ratio often dissociates from the relative ratio in cohorts including many patients with diseases. Considering the substantial numbers of participants with symptoms in the study, they should not handle the odds ratio as the relative risk.

For example (in the result section): “Health care providers whose age >40 years old were about eight times [AOR=7.983; 95% CI (1.443-44.174)], more likely to develop anxiety than health care providers whose age 18-25 years old”,

I agree that the difference is statistically significant, but the risk is not “eight times”. So, how about just changing like the below? “Health care providers whose age >40 years old were significantly more likely to develop anxiety than health care providers whose age 18-25 years old” [AOR=7.983; 95% CI (1.443-44.174)], Please also revise the many other descriptions regarding AOR.

**Response by the authors**

Dear reviewer,

Thank you for your constructive comments;

It is corrected based on your kind and constructive comment. Please kindly refer to page 19 line number 15-19, page 20 line 1-5, page 23 line 11-20, page 24 line 1-2 and page 27 line 13-18

2. Is the manuscript technically sound, and do the data support the conclusions?

The manuscript must describe a technically sound piece of scientific research with data that supports the conclusions. Experiments must have been conducted rigorously, with appropriate controls, replication, and sample sizes. The conclusions must be drawn appropriately based on the data presented.

Reviewer #1: Partly

**Response by the authors**

Dear reviewer,

Thank you for your constructive comments;

It is corrected based on the comment. Please kindly refer page 34 line 16-21 and page 35 line 1-2

***Yours sincerely,***

***Fisha Alebel GebreEyesus (MSc)*** *- (corresponding author) on behalf of all authors)*

*,*
